# Impact of an early education multimedia intervention in managing nutrition-related chemotherapy side effects: a pilot study

**DOI:** 10.1186/2193-1801-2-179

**Published:** 2013-04-23

**Authors:** Julie Thompson, Kathryn Silliman, Dawn E Clifford

**Affiliations:** Department of Nutrition and Food Sciences, California State University, Chico, CA 95929-0002 USA

**Keywords:** Cancer, Chemotherapy, Nutrition education, Side effects, DVD, Patient education

## Abstract

**Background:**

The purpose of the educational intervention was to measure changes in knowledge, perceived benefit of nutrition, and perceived self efficacy in handling side effects of chemotherapy before and after viewing a 15 minute DVD among patients with cancer.

**Methods:**

A convenience sample of 14 (4 male, 10 female, 61 ± 9 years) patients with cancer, early to chemotherapy, participated in the study. Participants completed a survey with demographic, knowledge items, and perceived health belief and self efficacy statements; viewed the DVD; and were then sent home with a one page handout. Two weeks after the nutrition education intervention, a second survey was completed including an item about tips used. Change was measured using paired *t*-test and wilcoxon signed rank tests.

**Findings:**

The mean score on the four knowledge items significantly increased (p < 0.05). No significant differences were noted in statements intended to evaluate changes in perceived health beliefs. However, perceived knowledge and skills related to managing side effects increased (p < 0.05). All participants responded that the DVD was informative and most (n = 11, 79%) responded that it was useful. The majority reported (n = 10, 71%) a tip they used from the DVD.

**Conclusions:**

This short multimedia nutrition education intervention was found primarily to increase knowledge and could form a useful component of counseling services for patients undergoing chemotherapy.

## Background

Chemotherapy is a common treatment for most types of cancer. Side effects of chemotherapy include nausea, vomiting, diarrhea, constipation, appetite change, anorexia, dysgeusia, and food aversions and lead to malnutrition (Capra et al. 2001). Managing the nutritional side effects helps reduce the risk of malnutrition which is associated with morbidity, mortality and lower quality of life (Capra et al. 2001; Caro et al. 2007). Prior research has shown that effective nutrition interventions are necessary during cancer treatment and lead to more positive outcomes (Baur and Capra 2005; Paccagnella et al. 2010, 2011; Ravasco et al. 2005, 2006).

A few studies have assessed cancer patients’ needs and preferences for education related to nutrition (Hartmuller and Desmond 2004; Lock and Wilson 2002; Piredda et al. 2008). Among the most important topics that patients want to learn about are side effects of treatment (Hartmuller and Desmond 2004; Piredda et al. 2008; Lock and Wilson 2002). Most patients want to receive information during treatment (Hartmuller and Desmond 2004) or as soon as they know they are going to be given chemotherapy (Piredda et al. 2008). The preferred method for receiving information about side effects of chemotherapy is oral conversation (Piredda et al. 2008) or one-on-one discussion with health professionals (Lock and Wilson 2002), followed by written information (Lock and Wilson 2002; Piredda et al. 2008). A needs assessment was conducted at the location of the current study with 50 patients with cancer (84% received chemotherapy) (Busby 2009). The majority (58%) indicated that receiving nutrition education while going through treatment was very important. However, the majority (63%) had not received any nutrition education from a health care provider, although 64% reported having a weight change in the past four months. When asked to rank the topics (from a list of eight) to include in educational materials for patients with cancer, the most frequently chosen topics were coping with nutritional side effects of treatment and eating a balanced diet. In addition, most patients (56%) wanted one-on-one counseling.

Due to limited resources, the health care team decided to develop a nutrition education DVD on the nutrition-related side effects of chemotherapy (as a substitute for one-on-one counseling) and a one page handout for patients to take home that highlighted the main points of the DVD. The Conceptual Model for Communicating Nutrition, a model for planning, implementing, and evaluating nutrition education programs (Gillespie and Yarbrough 1984); the Health Belief Model, where an individual is likely to take action towards better health if they are aware of severity of a disease (Rimer 2002); and the concept of self efficacy (Banduras 1977), which refers to a person’s belief in his or her ability to succeed in a particular situation, provided the framework for developing the DVD. The goal of the DVD and the handout was to assist cancer patients in preparing for side effects of chemotherapy. The purpose of the educational intervention was to measure change in knowledge, perceived benefit of nutrition, and perceived self efficacy in handling side effects.

## Methods

### Study design and participants

A convenience sample of 14 newly diagnosed patients with cancer were recruited from a rural outpatient cancer center, Enloe Regional Cancer Center. The study protocol was approved by the Enloe Institutional Review Board. Registered nurses (RNs) working in the infusion suite of the center reviewed patient lists to identify possible participants. Inclusion criteria included confirmed diagnosis of cancer, in the beginning to middle of chemotherapy treatment (the number of treatments received had to be less than the number of treatments to follow); at least 18 years of age; and ability to read and write in English. If the patient had trouble communicating or understanding the primary researcher [JT], they were excluded.

After participants provided written consent, they completed a survey (before intervention). Then the researcher set up the participants with a mini DVD player loaded with a 15-minute DVD titled, “Supportive Care: Managing Nutrition Related Side Effects during Chemotherapy.” After watching the DVD during their infusion treatment, they were given a one page handout to take home. Two weeks following the nutrition education intervention, participants were phoned by the researcher and a second survey was administered (after intervention). Five participants opted out of the phone call due to hearing impairment and completed the survey at the cancer center at the subsequent visit.

### Outcome measures & intervention

The survey was designed to assess the constructs of knowledge, perceived benefit of nutrition, and perceived self efficacy. The number of items was limited (n = 16) knowing that fatigue is a common side effect of chemotherapy. Six items addressed demographics, number of chemotherapy treatments, prior education received, dietary changes made since diagnosis, and prescribed diets. Four multiple choice items tested participants’ knowledge about the nutritional side effects of chemotherapy. The participants were given a list of five answers to each of four questions, including “I don’t know,” and was instructed to circle all of the correct responses; knowing that there may be more than one correct answer. Cronbach’s alpha coefficient for the knowledge items was .86. Four items assessed the patients’ health beliefs (perceived benefit on nutrition (one statement), perceived behavioral control (one statement), and perceived self efficacy (two statements). Cronbach’s alpha coefficient for these four items was .75. One item asked about the behavior of food avoidance due to side effects. Participants rated these items on a five-point Likert scale (1 = strongly disagree, 2 = disagree, 3 = have no opinion, 4 = agree, or 5 = strongly agree).

The after intervention survey had the same four knowledge questions and five health belief, perceived self-efficacy, and behavior statements as the before intervention survey. Four extra items were added to assess the usefulness of the nutrition education intervention and included the following. Two items, using the same five-point Likert scale, assessed whether the DVD was informative and provided knowledge about managing the nutritional side effects of chemotherapy treatment. A third item assessed whether the tips provided in the DVD were actually used. If participants said yes, then they were asked what tip they used. A fourth item assessed whether the participants would recommend the DVD to others undergoing chemotherapy.

The nutrition education intervention, the DVD, was designed by registered dietitians (RDs) and RNs from Enloe Regional Cancer Center and California State University, Chico. The script for the DVD was written and narrated by the researcher. The primary focus of the DVD was the common nutrition-related side effects of chemotherapy (weight loss, loss of appetite, nausea, vomiting, constipation, diarrhea, changes in taste and smell, and mouth or throat dryness and soreness). The tips in the video were adopted from the 2008 National Cancer Institute's pamplet “Eating Hints for Cancer Patients Before, During and After Treatment” (National Cancer Institute 2008). The side effects and tips were discussed by three RDs. The program host discussed the importance of good nutrition during chemotherapy. Two cancer survivors shared personal experiences pertaining to nutrition and chemotherapy. A one page handout was created to summarize the eight side effects and tips presented in the DVD.

### Analysis

Data were analyzed using the Statistical Package for Social Sciences (SPSS) version 18.0. Descriptive statistics were used to describe participant characteristics. For each knowledge item, there were three correct answers, one incorrect answer, and “I don't know/I'm not sure.” Each correct answer selected was worth one point and if the incorrect answer was not selected one additional point was awarded. Therefore, each item had four points possible. If no answers were selected, or “I don't know” was selected, or if the incorrect answer was the only answer selected no points were awarded for that item. Paired student’s *t*-test was used to analyze the change in knowledge score. Comparisons between participants responses to items that used a Likert-scale were evaluated using wilcoxon signed rank tests.

## Results and discussion

Four males and 10 females participated in the study (61 ± 9 years). They had the following cancer diagnoses: reproductive/breast (n = 6); lung (4); colorectal (2); other (2). The majority (57%) reported they had some college education. The average number of chemotherapy treatments at the time of intervention was 2.0 ± 1.5. Only 21% reported they had received dietary counseling prior to the intervention. The same number of participants (21%) reported that they were following a prescribed therapeutic diet. More than half of subjects (57%) reported making dietary changes after diagnosis. The score on all four knowledge items increased significantly two weeks after viewing the nutrition education DVD (Table 1). Thus, the results of this pilot study demonstrate that a DVD on nutrition-related side effects of chemotherapy along with a one-page handout increased knowledge in the short term.

**Table 1:** **The number of correct responses to knowledge items**

	Before	After	p value^a^
If I experience a loss of appetite during my chemotherapy treatments I should…	1.4 ± 1.3^b^	2.7 ± 1.3	.001
If I feel nausea during my chemotherapy treatments I should …	1.2 ± 1.1	2.1 ± 0.5	.03
If I am experiencing constipation during my chemotherapy treatments I should…	2.2 ± 1.5	3.5 ± 0.7	.01
If I am experiencing mouth and throat sores during my chemotherapy treatments I should…	1.5 ± 1.5	3.4 ± 0.5	.001

^a^paired *t*-test.

^b^mean ± SD number of correct responses out of 4 possible.

No significant changes were noted in statements intended to evaluate perceived beliefs about the importance of nutrition or belief about being responsible for managing side effects (Table 2). However, it should be noted that prior to watching the DVD most participants already agreed or strongly agreed that nutrition is important in managing side effects and that they were responsible for managing side effects. In addition, it appears that participants had not exhibited food avoidance behavior related to side effects of chemotherapy. It may be that participants had not yet experienced chemotherapy side effects.

**Table 2:** **Survey responses to health belief/perceived self efficacy statements**

	Before	After	Z statistic^a^	p value^a^
	Median (IQR)	Median (IQR)		
Nutrition is important in managing my side effects of chemotherapy.^c^	4 (1)^b^	5 (1)	−1.63	.10
To date, side effects of my treatment have caused me to avoid or limit some foods^d^	3 (2)	4 (1)	−1.27	.21
I am responsible for managing the nutrition related side effects of chemotherapy^e^	5 (1)	5 (1)	−0.58	.56
I have enough knowledge to manage the nutrition related side effects of chemotherapy^f^	2 (2)	4 (2)	−2.36	.02
I have the skills needed to manage the nutrition related side effects of chemotherapy^f^	3 (2)	4 (1)	−2.86	.004

^a^wilcoxon ranked sign tests.

^b^values are median and interquartile range (IQR); Likert scale:1 = Strongly disagree 2 = Disagree 3 = No opinion 4 = Agree 5 = Strongly agree.

^c^perceived benefit statement.

^d^perceived behavior statement.

^e^perceived behavioral control.

^f^perceived self efficacy statement.

Participants showed significant increases in perceived self efficacy after the nutrition education intervention compared with before. Higher agreement scores were reported for participants’ perceived knowledge and skills related to managing the side effects of chemotherapy. Thus, the DVD and handout may provide benefit in improving the participants’ self-confidence in coping with side effects. Our study is unique in that the focus of the educational intervention was nutrition and chemotherapy. Other researchers using multi-media CD-ROMs as the intervention, on cancer and treatment topics unrelated to nutrition, have reported similar findings. Studies with teens and children report that patients with cancer have greater feelings of control over their health (Dragone et al. 2002; Jones et al. 2010). Wydra (2001) reported that patients with cancer showed significant improvement in self-care ability compared to a control group. Schofield et al. (2008) developed a chemotherapy educational DVD, on preparation for chemotherapy and self-management of eight common side effects, that was shown to patients with cancer prior to initiation of chemotherapy. A second group of patients with cancer received usual care. Within these two groups the researchers also stratified the patients into self-perceived curative (believed they would be cured of cancer) and self-perceived palliative (did not believe). For the self-perceived curative group, those that watched the DVD had greater self-efficacy for social support than those that received usual care. For the self-perceived palliative group, those that watched the DVD were more satisfied with information about side effects than those receiving usual care.

The participants in this pilot study not only showed an increase in knowledge, but demonstrated application of knowledge by reporting tips they used. Two weeks after watching the DVD all patients agreed or strongly agreed that the DVD was informative and reported that they would recommend the DVD to others undergoing chemotherapy. The majority of participants (79%) reported that the video was useful and 71% were able to give one tip they had used. The most common tip they used was “eat small frequent meals throughout the day.” Other tips reported were, “eat a balanced diet,” “eat by the clock and not by hunger,” “ginger helps with nausea,” “get an adequate amount of protein,” “warm food exacerbates nausea,” and “what to do for diarrhea.”

There were limitations to this pilot study. The sample size was small and from a single site, follow-up was of a short time frame, and we were unable to assess nutritional status before or after the educational intervention to determine clinical outcomes. Also, the number of items on the survey was limited so results should be interpreted with caution. A larger study is needed to investigate the impact of an educational DVD of nutrition-related side effects of chemotherapy on changes in diet-related behavior, caloric and nutrient intake and clinical outcomes such as severity of side effects, weight, lean body mass, and quality of life.

## Conclusion

Maintaining good nutritional status is vital for patients with cancer. We found that only 21% of patients received diet counseling prior to enrolling in this study. Hartmuller and Desmond (2004) reported that almost half of patients with cancer did not receive dietary counseling from any healthcare professional. This pilot study has shown that a 15 minute educational DVD on nutrition-related side effects of chemotherapy leads to increased knowledge and participants’ perceived knowledge and skills related to managing the side effects of chemotherapy. An educational DVD, if effective, is useful and may help patients with cancer cope with side effects of chemotherapy as many patients with cancer do not receive any diet counseling.

## References

[CR1_239] Banduras A (1977). Self efficacy: Toward a unifying theory of behavioral change. Psychol Rev.

[CR2_239] Baur JD, Capra S (2005). Nutrition intervention improves outcomes in patients with cancer cachexia receiving chemotherapy- A pilot study. Support Care Cancer.

[CR3_239] Busby V (2009). Assessment of the perceived need and preferred mode of nutrition education by patients and staff at a cancer treatment center in northern California.

[CR4_239] Capra S, Ferguson M, Ried K (2001). Cancer: Impact of nutrition intervention outcome-nutrition issues for patients. Nutrition.

[CR5_239] Caro M, Laviano A, Pichard C (2007). Nutritional intervention and quality of life in adult oncology patients. Clin Nutr.

[CR6_239] Dragone MA, Bush PJ, Jones JK, Bearison DJ, Kamani S (2002). Development and evaluation of an interactive CD-ROM for children with leukemia and their families. Patient Educ Couns.

[CR7_239] Gillespie A, Yarbrough P (1984). A conceptual model for communicating nutrition. J Nutr Educ.

[CR8_239] Hartmuller VM, Desmond SM (2004). Professional and patient perspectives on nutritional needs of patients with cancer. Oncol Nurs Forum.

[CR9_239] Jones JK, Kamani SA, Bush PJ, Hennessy KA, Marfatia A, Shad AT (2010). Development and evaluation of an educational interactive CD-ROM for teens with cancer. Pediatr Blood Cancer.

[CR10_239] Lock KK, Wilson B (2002). Information needs of cancer patients receiving chemotherapy in an ambulatory-care setting. Can J Nurs Res.

[CR11_239] (2008). Eating hints: Before, during and after cancer treatment.

[CR12_239] Paccagnella A, Morello M, DaMosto MC, Baruffi C, Marcon ML, Gava A, Baggio V, Lamon S, Babare R, Rosti G, Giometto M, Boscolo-Rizzo P, Kiwanuka E, Tessarin M, Caregaro L, Marchiori C (2010). Early nutritional intervention improves treatment tolerance and outcomes in head and neck cancer patients undergoing concurrent chemoradiotherapy. Support Care Cancer.

[CR13_239] Paccagnella A, Morassutti I, Rosti G (2011). Nutritional intervention for improving tolerance in cancer patients. Curr Opin Oncol.

[CR14_239] Piredda M, Rocci L, Gualandi R, Petitti T, Vincenzi B, Marinis MG (2008). Survey on learning needs and preferred sources of information to meet these needs in Italian oncology patients receiving chemotherapy. Eur J Oncol Nurs.

[CR15_239] Ravasco P, Monteiro-Grillo I, Vidal PM, Camilo ME (2005). Dietary counseling improves patient outcomes: A prospective, randomized, controlled trial in colorectal cancer patients undergoing radiotherapy. J Clin Oncol.

[CR16_239] Ravasco P, Monteiro Grillo I, Camilo M (2006). Cancer wasting and quality of life react to early individualized nutritional counseling!. Clin Nutr.

[CR17_239] Rimer BK, Glanz K, Rimer BK, Lewis FM (2002). Health belief model. Health behavior and health education: Theory, research, and practice.

[CR18_239] Schofield P, Jefford M, Carey M, Thomson K, Evans M, Baravelli C, Aranda S (2008). Preparing patients for threatening medical treatments: effects of chemotherapy educational DVD on anxiety, unmet needs, and self- efficacy. Support Care Cancer.

[CR19_239] Wydra EW (2001). The effectiveness of a self-care management interactive multimedia module. Oncol Nurs Forum.

